# Incidence and outcome of asymptomatic deep vein thrombosis in critically ill patients: a prospective cohort study

**DOI:** 10.1186/cc14406

**Published:** 2015-03-16

**Authors:** R Echigoya, H Okamoto, H Uchino, A Kuriyama, N Tamura, K Sato, T Fukuoka

**Affiliations:** 1 Kurashiki Central Hospital, Okayama, Japan

## Introduction

Asymptomatic deep vein thrombosis (DVT) including catheter-related thrombosis (CRT) is an increasingly recognized disease entity in critically ill patients. However, the reported rate and outcome of DVT vary widely depending on study design, patient background and detecting method. The objective of this study is to evaluate the incidence and outcome of DVT in adult critically ill patients.

## Methods

This study is a prospective cohort study of patients admitted to a medical and surgical ICU from 1 July 2014 to 15 October 2014. All consecutive patients over 18 years of age and with expected ICU stay over 72 hours were included. Patients who had previous history of DVTs were excluded. We examined internal jugular vein, subclavian vein, axillary vein, brachial vein, femoral vein, superficial femoral vein, and popliteal vein, on ICU admission and within 48 hours after ICU discharge. The DVT was diagnosed using compression ultrasonography with color Doppler. Images were interpreted by two independent investigators trained in ultrasonography. All patients received intermittent pneumatic compression and unfractionated heparin twice daily during their IUC stay. Once the DVT was detected, therapeutic anticoagulation was initiated. Contrast-enhanced CT was performed when the patients were suspected to have pulmonary embolism. The primary outcome was the incidence of DVT during the ICU stay. Patients were followed until their hospital discharge.

## Results

A total of 51 patients were included. The median age and BMI were 73 years and 23 kg/m^2^, respectively; 31% were female and 69% were surgical critical care patients. The median APACHE II and SOFA scores were 20 and 8, respectively. Risk factors associated with DVT were presence of central venous catheter 63%, malignancy 9% and hemodialysis 14%. The rate of DVT was 18.6% and the rate of CRT was 13.7%. All of these were asymptomatic and seen in neck and upper extremities. There was no DVT-associated adverse event (pulmonary embolism, bleeding) during hospital stay. The 28-day all-cause mortality rate was 3.4%.

## Conclusion

While incidence of asymptomatic DVT is relatively high in adult critically ill patients, they were found only in the neck and upper extremities without any adverse event. Further research is needed to evaluate the clinical significance of this type of DVT.

